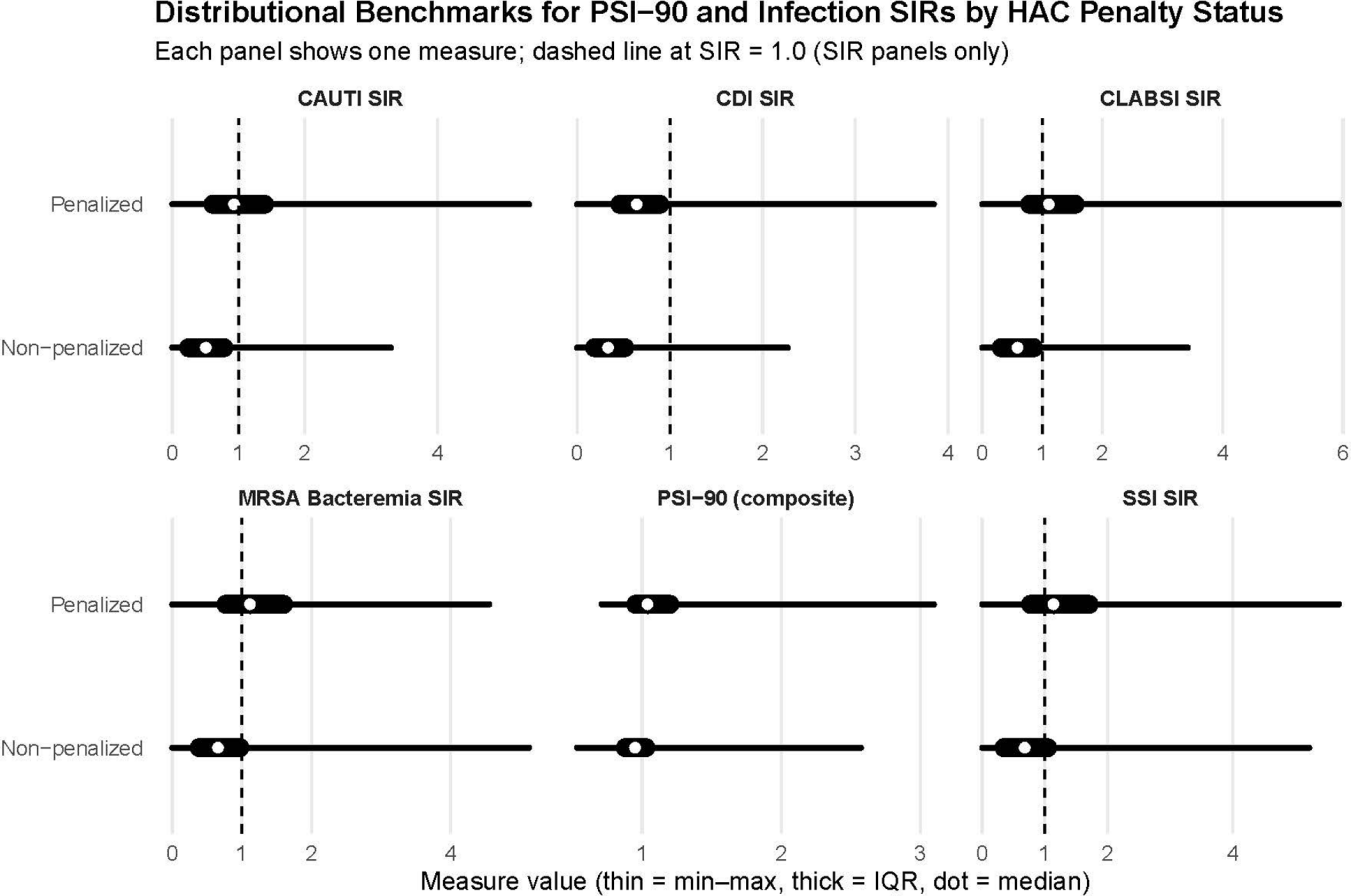# 361 Validation of an Internal Infection Control Chatbot

**DOI:** 10.1017/ash.2026.10697

**Published:** 2026-06-23

**Authors:** Kelly Holmes, Kelley Boston, Holly Taylor

**Affiliations:** 1 Infection Prevention & Management Associates; 2 Ascension Texas

## Abstract

**Background:** The Centers for Medicare & Medicaid Services (CMS) Hospital-Acquired Condition Reduction Program (HACRP) assigns penalties to the worst-performing quartile of U.S. hospitals based on the Patient Safety and Adverse Events Composite (PSI-90) and five standardized infection ratios (SIRs). Although these measures are weighted equally in program scoring, the extent to which each metric contributes to penalty risk is unclear. Understanding the relative influence of these measures is essential for infection preventionists (IPs) and quality leaders working to anticipate penalties and prioritize improvement efforts. **Methods:** We conducted a retrospective cross-sectional analysis of 3,082 acute care hospitals included in the CMS Fiscal Year (FY) 2025 HACRP dataset. Predictors included PSI-90 and SIRs for central line-associated bloodstream infection (CLABSI), catheter-associated urinary tract infection (CAUTI), surgical site infection (SSI), Clostridioides difficile infection (CDI), and methicillin-resistant Staphylococcus aureus (MRSA) bacteremia. All measures were analyzed as continuous variables and standardized using z-scores. Multivariable logistic regression estimated associations with penalty status. Model discrimination was assessed using area under the curve (AUC). **Results:** All six measures were strongly associated with HACRP penalties. PSI-90 demonstrated the largest effect size (OR 10.87; 95% CI: 7.76–15.7). Among infection-specific measures, CAUTI (OR 8.60; 95% CI: 6.27–12.1) and CDI (OR 8.48; 95% CI: 6.25–11.8) showed the strongest associations. The primary model exhibited excellent discrimination (AUC = 0.99). Distribution plots revealed that penalized hospitals consistently had higher PSI-90 and SIR values; for CAUTI and CDI, even small shifts toward or above an SIR of 1.0 increased penalty likelihood. **Conclusions:** In FY2025, PSI-90 demonstrated the strongest association with HACRP penalty assignment, despite equal weighting with infection-specific SIRs. Among infection measures, CAUTI and CDI showed the greatest influence on penalty risk. These findings provide actionable benchmarks for IPs and quality leaders to interpret performance and anticipate penalty risk within HACRP.

**Figure:**
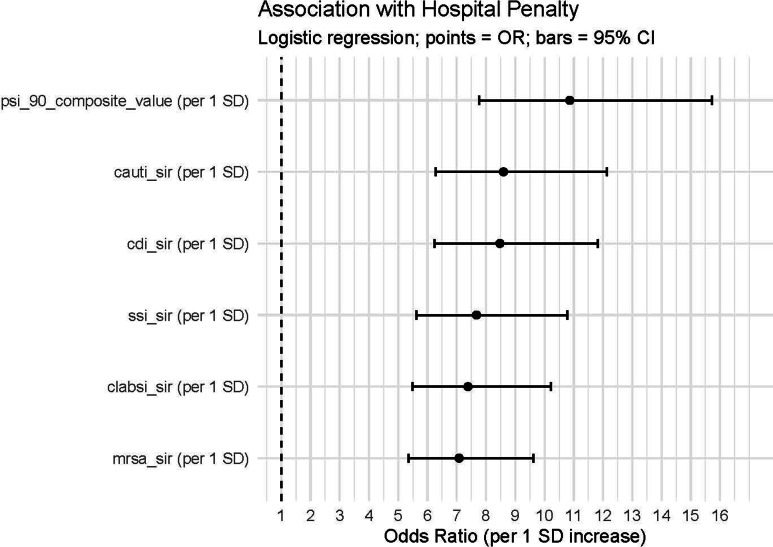

**Figure:**